# Editorial: The role of light in myopia

**DOI:** 10.3389/fmed.2026.1941493

**Published:** 2026-09-02

**Authors:** Natali Gutierrez-Rodriguez, Valentina Bilbao-Malavé, Manuel Garza-León, Sandra Johana Ganzón-Parra, Jorge González-Zamora, Sergio Recalde Maestre

**Affiliations:** 1 Grupo de Investigación Cuidado Primario Visual y Ocular, Facultad de Ciencias de la Salud, Universidad de La Salle, Bogotá, Colombia; 2 Retinal Pathologies and New Therapies Group, Experimental Ophthalmology Laboratory, Department of Ophthalmology, Clinica Universidad de Navarra, Pamplona, Spain; 3 Institut Clínic de Oftalmologia (ICOF), Hospital Clínic de Barcelona, Barcelona, Spain; 4 Science of Health Division, Clinical Science Department, University of Monterrey, San Pedro Garza García, Nuevo León, México; 5 Research Group in Optometry, Faculty of Optometry, Antonio Nariño University, Bogotá, Colombia; 6 Department of Ophthalmology, University Hospital of Navarra, Pamplona, Spain; 7 Institute for Health Research, Instituto de Investigación Sanitaria de Navarra (IdiSNA), Pamplona, Spain; 8 Department of Ophthalmology, Clínica Universidad de Navarra, Pamplona, Spain

**Keywords:** CUVAF, green space, light, myopia-epidemiology, outdoor, red light

Myopia has become one of the greatest challenges in vision health owing to its rapidly increasing global prevalence and the growing burden of vision-threatening complications associated with high myopia, including myopic maculopathy, glaucoma, retinal detachment, and cataract. Together, these conditions represent major causes of visual impairment and blindness in adulthood. Although genetic predisposition plays an important role in the development of myopia, the dramatic rise in its prevalence over recent decades highlights the substantial contribution of environmental factors. Among these, light exposure has emerged as one of the key modulators of ocular growth and the emmetropization process.

Research into the role of light in myopia has evolved considerably over the past two decades. Initial epidemiological studies demonstrated the protective effect of time spent outdoors against the onset of myopia, while subsequent advances in ocular biology identified physiological and molecular mechanisms that may explain this association. Current evidence indicates that not only light intensity but also its spectral composition, duration, and timing of exposure influence multiple signaling pathways involved in ocular growth regulation and refractive development.

This Research Topic, *The role of light in myopia*, brings together contributions that illustrate the breadth and rapid development of this field. The articles included examine light as an environmental determinant of myopia development and progression, explore the biological mechanisms underlying ocular growth regulation, present objective approaches for quantifying outdoor light exposure, and evaluate emerging therapeutic strategies based on light modulation.

From an epidemiological perspective, Li et al. demonstrated that specific childhood and adolescent lifestyle patterns are associated with protection against myopia, with greater outdoor exposure combined with reduced near-work activities providing the greatest protective benefit. Complementing these findings, Barnett-Itzhaki et al.

highlighted the importance of green spaces surrounding schools and urban environments, emphasizing how these environments promote outdoor activity while providing diverse visual stimuli at multiple viewing distances. Together, these studies reinforce the concept that environmental and urban planning should be considered an integral component of public health strategies aimed at reducing the global burden of myopia.

Although the protective role of outdoor exposure is now well-established, accurately quantifying individual light exposure remains a significant challenge. In this context, conjunctival ultraviolet autofluorescence (CUVAF) has emerged as a promising, non-invasive biomarker reflecting cumulative ultraviolet exposure. De La Puente et al. reported an inverse association between CUVAF area and the degree of myopia, supporting its potential as an objective marker of outdoor exposure, particularly in pediatric populations. Their findings are further expanded by subsequent work suggesting that CUVAF may not only help identify children at increased risk of developing myopia but also serve as an objective tool for monitoring adherence to outdoor-based preventive interventions.

Several contributions also deepen our understanding of the structural and biological changes accompanying myopia progression. Cao et al. investigated the relationship between axial length and retinal, choroidal, and vascular alterations in children and adolescents, providing evidence that the deep retinal microvasculature is particularly susceptible to axial elongation. Meanwhile, Singh and Martin reviewed the growing evidence supporting the role of different light wavelengths, photobiomodulation, and circadian regulation in emmetropization, emphasizing the complex biological pathways through which light influences ocular development.

Light-based therapeutic interventions represent one of the fastest-growing areas of contemporary myopia research. In particular, repeated low-level red-light therapy has attracted considerable attention because of its promising clinical outcomes. Liu et al., through a systematic review and meta-analysis, demonstrated that this treatment is associated with reduced spherical equivalent progression, slower axial elongation, and increased choroidal thickness. Similarly, Ren et al. explored its effects on corneal curvature, axial length, and refractive error, proposing that selective stimulation of rod and cone pathways may contribute to modifying ocular growth. Although these findings are encouraging, both studies emphasize the need for well-designed longitudinal investigations to establish long-term efficacy and safety. In the same direction, Yang et al. showed that combining 650-nm red-light therapy with distance-image therapy may further enhance choroidal thickening while reducing axial elongation in children, reinforcing the therapeutic potential of light-based interventions.

Finally, Murphy et al., using an experimental chick model, provide novel insights into the role of Müller glial cell-mediated ion and fluid transport during light-dark cycles. Their findings suggest previously unrecognized physiological mechanisms involved in ocular growth regulation and identify potential pharmacological targets that may complement future strategies for myopia control.

Collectively, the studies presented in this Research Topic underscore the central role of light in the prevention, development, and treatment of myopia. They strengthen the evidence supporting outdoor exposure as one of the most cost-effective interventions for reducing childhood myopia incidence, highlight the potential of CUVAF as an objective biomarker of light exposure, and illustrate the growing interest in light-based therapeutic approaches, particularly low-level red-light therapy. At the same time, these contributions expand our understanding of the biological mechanisms regulating ocular growth while identifying new research directions and therapeutic opportunities. Nevertheless, important questions remain regarding the precise mechanisms by which light influences ocular development and the long-term efficacy and safety of emerging light-based interventions across different populations.

